# Plant Genetics as a Tool for Manipulating Crop Microbiomes: Opportunities and Challenges

**DOI:** 10.3389/fbioe.2021.567548

**Published:** 2021-05-31

**Authors:** Kayla M. Clouse, Maggie R. Wagner

**Affiliations:** ^1^Department of Ecology and Evolutionary Biology, University of Kansas, Lawrence, KS, United States; ^2^Kansas Biological Survey, University of Kansas, Lawrence, KS, United States

**Keywords:** microbiome manipulation, crop microbiome, plant genetics, sustainable agriculture, genetic engineering, breeding, plant-microbe interactions

## Abstract

Growing human population size and the ongoing climate crisis create an urgent need for new tools for sustainable agriculture. Because microbiomes have profound effects on host health, interest in methods of manipulating agricultural microbiomes is growing rapidly. Currently, the most common method of microbiome manipulation is inoculation of beneficial organisms or engineered communities; however, these methods have been met with limited success due to the difficulty of establishment in complex farm environments. Here we propose genetic manipulation of the host plant as another avenue through which microbiomes could be manipulated. We discuss how domestication and modern breeding have shaped crop microbiomes, as well as the potential for improving plant-microbiome interactions through conventional breeding or genetic engineering. We summarize the current state of knowledge on host genetic control of plant microbiomes, as well as the key challenges that remain.

## Introduction

As the human population grows, food production must increase accordingly despite global climate change presenting more challenging conditions for crop production. Conventional agriculture has been largely successful at maintaining yields while adapting to environmental challenges. However, it has also caused environmental degradation, including non-point source pollution, declines in soil productivity, pesticide resistance, and reduced genetic diversity. Recently, plant microbiomes have received growing attention as possible tools for sustainable agriculture. A wide range of microbiome benefits have been reported, including protection against pathogen and pests (van Wees et al., 2008; Pineda et al., 2010), increased tolerance to drought and nutrient stress (Meena et al., 2017), changes in flowering time (Wagner et al., 2014; Lu et al., 2018), enhanced plant productivity (Berendsen et al., 2012), and heavy metal immobilization (Di Gregorio et al., 2006; Chatterjee et al., 2009). Based on our current understanding and available technologies, several approaches for crop microbiome manipulation already exist – the most common being manipulation through soil management techniques (Hartman et al., 2018; Nelkner et al., 2019) and inoculation with beneficial microbes (Parnell et al., 2016). Here, we suggest that manipulation of the host genome should be considered as another approach to manipulating crop microbiomes.

Next-generation sequencing technologies and culture-dependent methods have advanced the field of microbiome engineering, especially the production of microbial inoculants. The application of microbial inoculants is intended to increase a target function, such as vigor or biocontrol activities; however, their success is highly variable with few inoculants efficiently translated from greenhouse to field (Fukami et al., 2016; Kecskés et al., 2016; Mitter et al., 2017). Some causes of field failure may reside in the establishment and functionality of microbials. A successful inoculant must be able to establish in the environment, which is often challenging due to intense competition with indigenous microorganisms. Furthermore, the functionality of the inoculant may be altered or disrupted by environmental conditions, timing of inoculation, and microbial evolution (Kaminsky et al., 2019). One potential method to circumvent these limitations of microbial inoculants is to alter host genetics to promote the recruitment and growth of beneficial microbes.

Decades of evidence have established that plants change the abiotic and biotic properties of soils, which in turn impact plant performance and agronomically important traits—a process known as plant-soil feedback (PSF; Bever et al., 2012; van der Putten et al., 2013). These feedbacks may have either positive or negative consequences for the plant. In agricultural systems, negative PSF causes nutrient depletion and the build-up of host-specific soil pathogens, which can be mitigated through crop rotation and intercropping (McDonald and Stukenbrock, 2016). Positive PSF is also common in both natural and agricultural systems due to changes in host-specific symbiont density: helpful symbionts (e.g., mycorrhizal fungi and N-fixing bacteria) increase plant fitness, which in turn increases the abundance of a suitable host for those symbionts (O’Brien et al., 2021). While the theory underlying PSF experiments has mostly focused on a single pathogen or symbiont, the contribution of the complete soil microbiome to PSF in agricultural contexts is largely unknown (Dias et al., 2015). Nevertheless, because the PSF framework focuses on the role of the plant in shaping the microbial environment, it provides a solid foundation for understanding how host genotype can be leveraged to improve crop microbiomes.

Wild plants have selectively assembled microbiomes over their evolutionary histories, which have been disrupted by crop domestication and subsequent breeding (Gopal and Gupta, 2016). By selecting for yield in the context of high water and fertilizer inputs, conventional breeding has reduced the selection pressure to form beneficial microbial interactions. As a result, modern cultivars may have lost some genetic features required for the recruitment and management of specific microorganisms (Pérez-Jaramillo et al., 2016). For instance, domesticated broad beans were able to host fewer than half as many rhizobial strains as their wild progenitors (Mutch and Young, 2004), and intensive breeding has reduced the ability of soybeans to exclude ineffective “cheater” rhizobia (Kiers et al., 2007). Other studies have demonstrated distinct differences in the microbial community composition between wild and domesticated cultivars of wheat (Tkacz et al., 2020) and maize (Brisson et al., 2019). However, as with many other examples of host genotype influencing microbiomes, the functional consequences are unclear (see section “Predicting functional effects of microbiome variation” below). In general, the evidence that wild relatives consistently harbor superior microbiomes is mixed (Sawers et al., 2018) but wild relatives could serve as a reservoir of genetic variants if traits pertaining to beneficial microbial interactions are found.

Engineering microbiomes via the host genome has a major advantage over current microbial products in that it does not require changes to infrastructure or management, since plant breeding and genetic improvement is already common practice (Bakker et al., 2012). In conventional breeding, new varieties are developed by selectively crossing genotypes with complementary traits. Advancements in plant molecular biology have led to the development of modern breeding tools, beginning with transgenic approaches (Moose and Mumm, 2008). While transgenic methods transfer desirable traits from different species to a target species, it is now possible to directly “edit” the target species genome. The CRISPR-Cas system allows extremely precise changes to the plant genome, to modify traits concerning productivity, fitness, and biotic and abiotic stress management (Chen et al., 2019). Improvements to the CRISPR-Cas system (Varshney et al., 2015) are poised to make genome editing one of the preferred methods for crop improvement. All of these methods all have potential for optimizing crop microbiomes, although gene editing is likely more feasible than conventional breeding due to limitations in phenotyping microbiomes (described below). However, despite the historical success and recent advancements in plant genetic improvement, serious challenges must be addressed before implementing these methods for microbiome engineering. Here, we summarize the current state of knowledge on how plant genotypes affect the microbiome and outline some major obstacles that remain before host genetic improvements can be used to optimize crop microbiomes.

## Mechanisms of Host Genetic Control of the Microbiome

To date, most work on host genetic control of the microbiome has demonstrated differences in microbiome composition among cultivars or accessions with no well-defined genetic relationship to each other, often chosen to represent a breadth of diversity within the host species (Lundberg et al., 2012; Peiffer et al., 2013; Edwards et al., 2015; Wagner et al., 2016; Wallace et al., 2018; Walters et al., 2018; Liu et al., 2019; Veach et al., 2019). A minority of examples have specifically addressed the effects of domestication (Pérez-Jaramillo et al., 2017; Brisson et al., 2019) or modern breeding (Kiers et al., 2007; Wagner et al., 2020a,b). By demonstrating microbiome heritability, these studies suggest that crop microbiomes may have been indirectly shaped by domestication and selection, and could be further modified through additional conventional breeding. However, they are less informative about which specific host traits or genes could be targeted for their ability to improve the microbiome. In contrast, mutant and transgenic studies have revealed key genes and traits that govern the recruitment and regulation of the microbiome.

### Microbiome Recruitment

Because most microbiome members derive from the host’s immediate surroundings (Bulgarelli et al., 2012; Lundberg et al., 2012), plant genes that shape the host-environment interface—the traits separating the plant body from the ambient community of potential colonists, such as external root structures—have frequently been implicated in microbiome formation. In barley, for example, a single mutation altering the size and density of root hairs decreased microbial alpha diversity across two different soil types (Robertson-Albertyn et al., 2017). During domestication of common bean, changes in belowground morphology, including specific root length and root density, were associated with a decrease in the ratio of Bacteroidetes to Actinobacteria, among other changes to the bacterial rhizobiome (Pérez-Jaramillo et al., 2017). Aboveground, trichomes can serve as points of entry for some endophytes (Bailey et al., 2009) while also secreting antimicrobial compounds that may selectively inhibit others (Ramirez et al., 2012). Accordingly, genes underlying trichome development were overrepresented in candidate genes from a genome-wide association study of leaf microbiome composition in Arabidopsis (Horton et al., 2014). Mutations affecting the thickness and permeability of the leaf cuticle have also been demonstrated to affect leaf microbiome composition and, in turn, the microbiome’s ability to resist pathogen invasion (Reisberg et al., 2013; Bodenhausen et al., 2014; Ritpitakphong et al., 2016).

In addition to plant morphology defining the available microbial habitat, chemical modification of the host-environment interface also affects recruitment of beneficial microbes. Roots exude a range of molecules, including sugars and signaling compounds, that alter soil chemistry and provide nutrients for resident microbes (Lareen et al., 2016; Zhalnina et al., 2018). These exudates can shape the rhizosphere environment to selectively favor certain microbial groups (Broeckling et al., 2008). For example, transgenically-induced production of glucosinolate defensive chemicals in Arabidopsis shifted the relative abundances of Rhizobiaceae and other potentially important bacteria, demonstrating that gain-of-function genetic modification can be used to tailor the microbiome (Bressan et al., 2009). Similarly, in a loss-of-function mutant study, an antimicrobial coumarin selectively suppressed fungal pathogens in the rhizosphere while allowing colonization of growth-promoting bacteria (Stringlis et al., 2018). In another striking example, maize mutants that were deficient in the production of benzoxazinoids—defensive phytochemicals released by the roots of cereal crops—strongly shifted the composition of bacterial and fungal communities in the rhizosphere. This genetically-induced alteration to the soil microbiome had a durable effect on the growth and herbivore resistance of the next generation of plants, even after a winter fallow season (Hu et al., 2018). One of the most dramatic examples of host genotype facilitating a beneficial microbial association is the case of a maize landrace that exudes a thick, sugar-rich mucus from aerial roots, providing nutrition and habitat for a consortium of bacteria that in turn fix atmospheric nitrogen for the plant (Van Deynze et al., 2018). These cases illustrate the enormous potential of phytochemical variation to shape the microbiome, although more research is needed to fully understand the consequences for plant health.

### Maintenance and Regulation of the Microbiome

After the initial recruitment, plants are able to regulate their microbiomes through a variety of mechanisms including resource allocation and immune activity. In the well-studied legume-rhizobia mutualism, for example, plants can reduce the amount of carbon that they provide to bacterial strains that have colonized root nodules but do not provide nitrogen in return (Kiers et al., 2007). Arabidopsis thaliana uses a similar sanctioning strategy to deprive pathogenic bacteria of sugar, via a signal transduction pathway linking pattern recognition receptor (PRR) genes to phosphorylation of the gene sugar transport protein 13 (Yamada et al., 2016). Some of the same traits involved in microbiome recruitment are dynamically expressed by the plant to fine-tune their microbiomes; for instance, plants can quickly increase the production or degradation of secondary phytochemicals in response to a wide variety of stimuli, including pathogen attack or changes in nutrient status (Metlen et al., 2009).

Microbial growth and activity is also directly regulated by the plant immune system. The immune response begins with the activation of PRRs, which can detect a variety of highly conserved microbe-associated molecular patterns (MAMPs) such as flagellin and components of fungal and bacterial cell walls (e.g., chitin, peptidoglycans) (Zipfel, 2014). For this reason, PRR genes are a promising target for improving broad-spectrum disease resistance (Boutrot and Zipfel, 2017) and are also likely to affect microbiome assembly because MAMPs are conserved in non-pathogenic microbes. Similarly, both pathogens and non-pathogenic microbes produce effector molecules that can disarm or modulate the plant immune response (Rovenich et al., 2014; Nelson et al., 2017). Therefore, plant responses require precise coordination to protect against pathogens while allowing colonization by innocuous or potentially beneficial symbionts (Zipfel and Oldroyd, 2017; Rey and Jacquet, 2018). Indeed, multiple studies have shown that immune signaling gene pathways are involved in microbial symbiosis (Lebeis, 2014; Castrillo et al., 2017; Chen et al., 2020). Mutations that disrupted the biosynthesis and downstream targets of salicylic acid—a critical phytohormone that regulates the plant immune response—selectively altered rates of colonization by microbes from particular families, mostly in the Actinobacteria or Proteobacteria (Lebeis et al., 2015). Plant-commensal microbes feature a high diversity of MAMPs that vary in their ability to trigger immune responses, suggesting that precise modification of host genomes to regulate particular organisms might one day be possible (Colaianni et al., 2021).

Together, these studies demonstrate that modification of just one or a few genes in the host plant can profoundly impact the composition and function of the associated microbiome. These effects on plant-microbiome interactions should therefore be considered when developing new cultivars with desirable traits. However, several key challenges will have to be overcome before genetic modification of crop plants can be used to precisely fine-tune the microbiota for enhanced productivity.

## Challenges for Engineering Plant Microbiomes via Host Genotype

### “Phenotyping” the Microbiome

The invisibility and immense complexity of plant microbiomes present an immediate challenge for conventional breeding, which requires the ability to measure traits of interest quickly and inexpensively for very large numbers of plants. The current least expensive way to quantify microbiomes is via high-throughput amplicon sequencing; yet this approach quickly becomes cost-prohibitive at the scale needed for conventional breeding. Thus, barring major technological advancements that reduce these costs, it is unlikely that microbiome properties *per se* (e.g., alpha diversity or the abundance of a particular taxon) can be successfully targeted through conventional breeding. This is particularly true given that selection for microbiome properties would likely reduce the efficiency of breeding for more accessible traits such as yield or disease resistance.

An alternative approach would be to select on variation in plant responses to microbes, which would not require quantification of the microbiome itself. Indeed, it is plausible that selection on emergent plant traits that are responsive to microbes has already indirectly and unintentionally shaped variation in crop-microbe interactions (Mueller and Sachs, 2015). However, identifying such genetic improvements to the microbiome (as opposed to direct effects on plant traits) would require expensive *post hoc* sequencing or subsequent experimentation. Recently, Ramírez-Flores et al. (2020) took an innovative approach to this problem by creating a maize mapping population that segregated for a loss-of-function mutation that rendered the plant unable to form a symbiotic relationship with mycorrhizal fungi. Thus, they were able to use standard QTL-mapping approaches to identify genetic variants for beneficial responses to mycorrhizae in a large breeding population. In general, however, the combined complexity of plant genomic variation and microbiome variation create a daunting challenge for microbiome optimization.

### Predicting Functional Effects of Microbiome Variation

Even if microbiomes could be phenotyped in a cost-effective way for whole breeding populations, translating microbiome composition data into actionable understanding of community function presents its own considerable challenge. The usefulness of amplicon sequencing for understanding microbiome function is limited both by the inability of barcoding genes to resolve taxa at the strain level, and by a lack of information about the metabolic abilities and behaviors of those taxa. Despite tremendous progress in the description of the plant microbiome, more fundamental studies are needed to determine how community composition translates into function. This is particularly important for agriculture, since some microorganisms have been shown to modulate abiotic and biotic stress, but the role of the rest of the community is unknown. Addressing microbiome function is challenging and will require multifaceted approaches, including advancements in experimental design, microbiome characterization, and modeling (Lebeis, 2014; Vorholt et al., 2017).

One promising experimental approach is the use of synthetic communities of culturable microbes that can be recombined into consortia to observe their interactions and effects on the host (Vorholt et al., 2017). Such studies allow for reproducible conditions and the ability to determine which microbes cause changes in plant phenotype. Synthetic community approaches have been used successfully to demonstrate the roles of host genes on microbiome assembly in Arabidopsis leaves (Bodenhausen et al., 2014), and to reveal microbe-microbe interactions that underlie stability and function of the maize root microbiome (Niu et al., 2017). Such studies are typically low-throughput and limited to readily-cultured microbes. Nevertheless, they are a powerful tool for dissecting causal relationships between plant genotype, microbiome composition, and functional outcomes such as host health and community stability.

Another way to learn about microbiome function is to go beyond amplicon sequencing (which only measures community composition, and cannot distinguish between closely related strains) by incorporating multi-omics methods. Because the same functions can be fulfilled by more than one organism (Louca et al., 2018), this functional redundancy makes it difficult to interpret observed changes in microbiome composition based on taxonomic composition alone. In contrast, shotgun metagenomics allows direct observation of microbial gene frequencies, and thus the functional potential of a given community. Metatranscriptomics, metaproteomics, and metabolomics come even closer to describing community function, because they provide information about microbial gene expression and metabolic activity (Malik et al., 2020). The downsides to these technologies include higher costs and incomplete reference genome databases that are insufficient for annotating a large proportion of such datasets (Breitwieser et al., 2019). Advancements in sequencing technology, combined with continued research into microbial genomic and functional diversity, will chip away at these limitations over time.

Finally, improved modeling methods will assist with translating microbiome composition to microbiome function. Often, these involve innovative computational tools to derive new insights from large multi-omics datasets that are not amenable to standard statistical approaches (Sankaran and Holmes, 2019). Recent developments in this area have largely come from researchers working on human microbiomes, who face similar challenges in inferring function from massively complex datasets. For example, information about the relative abundance and metabolic abilities of gut microbiome members can be combined to simulate how a given community will respond to changes in nutrient availability (Magnúsdóttir and Thiele, 2018). Ultimately, multi-omics data and modeling will reveal features of the metagenome, metatranscriptome, or metabolome that can be traced back to plant genes and traits that control microbiome recruitment or regulation. Such features represent targets for microbiome optimization that are both malleable through genetic modification of the host and more likely to have important functional effects.

### Genotype-by-Environment Interactions

Although the studies summarized above demonstrate the potential for genetic modification of plants to alter microbiomes in useful ways, most of them report results from experiments performed in laboratory conditions. It is unclear whether the same effects would be observed across the range of complex environments that plants encounter in the field. Most complex plant traits exhibit some degree of interaction between genotype and environment (GxE), reflecting genetic variation for phenotypic plasticity. Because plant phenotypes influence colonization and establishment by different microbes, plant microbiomes also reflect this GxE. However, because plant-associated microbiomes are derived from the environmental pool of potential colonists, they are shaped by an additional source of environmental variation: abiotic factors that influence microbial biogeography, such as soil pH (Fierer and Jackson, 2006). Finally, a given microbe’s effect on the host plant is often sensitive to mutualistic or antagonistic interactions with neighboring microbes (Niu et al., 2017; Durán et al., 2018). For these reasons, host genotype effects on the microbiome are particularly likely to be environment-dependent (Wagner et al., 2016).

Indeed, most studies of microbiome composition in complex environments have observed that GxE effects are at least as strong as any main effect of genotype (Peiffer et al., 2013; Wagner et al., 2016, 2020a; Walters et al., 2018). Partly due to strong environmental effects, the estimated heritability of whole-community diversity or composition is generally low (<5%) (Walters et al., 2018; Wagner et al., 2020b). Low heritability seems likely to be a major constraint on using conventional breeding to engineer microbiomes; however, it is possible that functionally important members of the microbiome—most of which are currently unknown—may be more heritable than the community as a whole (Wallace et al., 2018). In addition to GxE shaping plant microbiomes, the environment also determines the level of benefit provided by a given microbiome. For example, bacterial strains that conferred drought resistance to grapevine and peppers showed no beneficial effects under well-watered conditions, indicating that their benefits are specific to a particular environmental stress (Rolli et al., 2015). In addition, their growth-promoting abilities were stronger in plants with drought-sensitive root genotypes relative to drought-resistant genotypes. As another example, some mycorrhizal fungi may act either as mutualists or parasites depending on plant genotype and the chemical, physical and biotic state of the environment (Johnson et al., 1997). Despite the complications introduced by GxE, the existence of relatively stable “core microbiomes” across diverse environments offers some encouragement that plants already have a strong ability to select their microbiomes in predictable ways. For example, the root microbiomes of Arabidopsis plants grown in different soils were still more similar in composition to each other than to the original soils (Lundberg et al., 2012). The same was true for root microbiomes of 31 tropical plant species from diverse phyla across six distinct soils (Yeoh et al., 2017). This robustness makes members of the core plant microbiome high-priority targets for agricultural applications (Busby et al., 2017).

GxE is a classic and widespread challenge for plant breeders working to improve almost any trait (Falconer, 1952), and to some extent, existing methods to overcome it can also be applied to efforts to improve crop microbiomes. The adaptability and phenotypic stability of a genotype across environments are important considerations when developing new cultivars. By adapting existing genomic selection methods, plant breeders can account for GxE interactions and select cultivars with optimal performance either across environments or in specific environments (Burgueño et al., 2012; Jarquín et al., 2014; Roorkiwal et al., 2018). Such methods will indirectly favor alleles that promote beneficial interactions with the microbes in a given environment (Mueller and Sachs, 2015; Mueller et al., 2019).

## Conclusion

The improvement of crop microbiomes via genetic manipulation of the host is a promising approach because it has the potential to promote plant-driven enrichment of beneficial microbes from any environment and any soil community. In addition, it requires no additional resources or inputs, and avoids any unforeseen consequences of introducing new organisms into established agroecosystems. However, such methods are likely a long way in the future. A great deal of research is needed to provide the scientific foundation that would enable actionable genetic improvement strategies with predictable outcomes for microbiome function. We are currently limited by our understanding of both (1) how plant genetic variation maps onto microbiome variation, and (2) what features of the microbiome should be modified to make it “beneficial” for a given plant in a given environment. Even when both of these questions are answered in a given system, the immense complexity of natural microbiomes can mask compelling effects that were seen in a laboratory setting (Weinhold et al., 2018). In the short term, other applications of host genetic variation are more likely to be successful: namely, focusing on improving plants’ phenotypic responses to the microbiomes they are likely to encounter in farm soils. Ultimately, genetic improvement should be considered as one more tool for incorporating microbes into sustainable agriculture, which could be used in concert with other approaches such as soil management and microbial inoculants.

## Author Contributions

KC and MW conducted the literature search and wrote the article. Both authors contributed to the article and approved the submitted version.

## Conflict of Interest

The authors declare that the research was conducted in the absence of any commercial or financial relationships that could be construed as a potential conflict of interest.

## References

[B1] Bailey B. A. Strem M. D. Wood D. (2009). Trichoderma species form endophytic associations within Theobroma cacao trichomes. *Mycol. Res.* 113 1365–1376. 19765658 10.1016/j.mycres.2009.09.004

[B2] Bakker M. G. Manter D. K. Sheflin A. M. Weir T. L. Vivanco J. M. (2012). Harnessing the rhizosphere microbiome through plant breeding and agricultural management. *Plant Soil* 360 1–13. 10.1007/s11104-012-1361-x

[B3] Berendsen R. L. Pieterse C. M. J. Bakker P. A. H. M. (2012). The rhizosphere microbiome and plant health. *Trends Plant Sci.* 17 478–486. 22564542 10.1016/j.tplants.2012.04.001

[B4] Bever J. D. Platt T. G. Morton E. R. (2012). Microbial population and community dynamics on plant roots and their feedbacks on plant communities. *Annu. Rev. Microbiol.* 66 265–283. 10.1146/annurev-micro-092611-150107 22726216 PMC3525954

[B5] Bodenhausen N. Bortfeld-Miller M. Ackermann M. Vorholt J. A. (2014). A synthetic community approach reveals plant genotypes affecting the phyllosphere microbiota. *PLoS Genet.* 10:e1004283. 10.1371/journal.pgen.1004283 24743269 PMC3990490

[B6] Boutrot F. Zipfel C. (2017). Function, discovery, and exploitation of plant pattern recognition receptors for broad-spectrum disease resistance. *Annu. Rev. Phytopathol.* 55 257–286. 28617654 10.1146/annurev-phyto-080614-120106

[B7] Breitwieser F. P. Lu J. Salzberg S. L. (2019). A review of methods and databases for metagenomic classification and assembly. *Brief Bioinform.* 20 1125–1136. 10.1093/bib/bbx120 29028872 PMC6781581

[B8] Bressan M. Roncato M.-A. Bellvert F. Comte G. Haichar F. Z. Achouak W. (2009). Exogenous glucosinolate produced by Arabidopsis thaliana has an impact on microbes in the rhizosphere and plant roots. *ISME J.* 3 1243–1257. 10.1038/ismej.2009.68 19554039

[B9] Brisson V. L. Schmidt J. E. Northen T. R. Vogel J. P. Gaudin A. C. M. (2019). Impacts of maize domestication and breeding on rhizosphere microbial community recruitment from a nutrient depleted agricultural soil. *Sci. Rep.* 9:15611. 10.1038/s41598-019-52148-y 31666614 PMC6821752

[B10] Broeckling C. D. Broz A. K. Bergelson J. Manter D. K. Vivanco J. M. (2008). Root exudates regulate soil fungal community composition and diversity. *Appl. Environ. Microbiol.* 74 738–744. 10.1128/AEM.02188-07 18083870 PMC2227741

[B11] Bulgarelli D. Rott M. Schlaeppi K. Ver Loren van Themaat E. Ahmadinejad N. Assenza F. (2012). Revealing structure and assembly cues for Arabidopsis root-inhabiting bacterial microbiota. *Nature* 488 91–95. 22859207 10.1038/nature11336

[B12] Burgueño J. de los Campos G. Weigel K. Crossa J. (2012). Genomic prediction of breeding values when modeling genotype × environment interaction using pedigree and dense molecular markers. *Crop Sci.* 52 707–719. 10.2135/cropsci2011.06.0299

[B13] Busby P. E. Soman C. Wagner M. R. Friesen M. L. Kremer J. Bennett A. (2017). Research priorities for harnessing plant microbiomes in sustainable agriculture. *PLoS Biol.* 15:e2001793. 10.1371/journal.pbio.2001793 28350798 PMC5370116

[B14] Castrillo G. José Pereira P. Paredes S. H. Law T. F. de Lorenzo L. Feltcher M. E. (2017). Root microbiota drive direct integration of phosphate stress and immunity. *Nature* 543 513–518. 28297714 10.1038/nature21417 PMC5364063

[B15] Chatterjee S. Sau G. B. Mukherjee S. K. (2009). Plant growth promotion by a hexavalent chromium reducing bacterial strain, Cellulosimicrobium cellulans KUCr3. *World J. Microbiol. Biotechnol.* 25 1829–1836. 10.1007/s11274-009-0084-5

[B16] Chen K. Wang Y. Zhang R. Zhang H. Gao C. (2019). CRISPR/Cas genome editing and precision plant breeding in agriculture. *Annu. Rev. Plant Biol.* 70 667–697. 10.1146/annurev-arplant-050718-100049 30835493

[B17] Chen T. Nomura K. Wang X. Sohrabi R. Xu J. Yao L. (2020). A plant genetic network for preventing dysbiosis in the phyllosphere. *Nature* 580 653–657. 32350464 10.1038/s41586-020-2185-0 PMC7197412

[B18] Colaianni N. R. Parys K. Lee H.-S. Conway J. M. Kim N. H. Edelbacher N. (2021). A complex immune response to flagellin epitope variation in commensal communities. *Cell Host Microbe* 29 635.e9–649.e9. 10.1016/j.chom.2021.02.006 33713602

[B19] Di Gregorio S. Barbafieri M. Lampis S. Sanangelantoni A. M. Tassi E. Vallini G. (2006). Combined application of Triton X-100 and Sinorhizobium sp. Pb002 inoculum for the improvement of lead phytoextraction by Brassica juncea in EDTA amended soil. *Chemosphere* 63 293–299. 10.1016/j.chemosphere.2005.07.020 16153689

[B20] Dias T. Dukes A. Antunes P. M. (2015). Accounting for soil biotic effects on soil health and crop productivity in the design of crop rotations. *J. Sci. Food Agric.* 95 447–454. 10.1002/jsfa.6565 24408021

[B21] Durán P. Thiergart T. Garrido-Oter R. Agler M. Kemen E. Schulze-Lefert P. (2018). Microbial interkingdom interactions in roots promote arabidopsis survival. *Cell* 175 973.e14–983.e14. 10.1016/j.cell.2018.10.020 30388454 PMC6218654

[B22] Edwards J. Johnson C. Santos-Medellín C. Lurie E. Podishetty N. K. Bhatnagar S. (2015). Structure, variation, and assembly of the root-associated microbiomes of rice. *Proc. Natl. Acad. Sci. U.S.A.* 112 E911–E920. 10.1073/pnas.1414592112 25605935 PMC4345613

[B23] Falconer D. S. (1952). The problem of environment and selection. *Am. Naturalist* 86 293–298. 10.1086/281736

[B24] Fierer N. Jackson R. B. (2006). The diversity and biogeography of soil bacterial communities. *Proc. Natl. Acad. Sci. U.S.A.* 103 626–631. 16407148 10.1073/pnas.0507535103 PMC1334650

[B25] Fukami J. Nogueira M. A. Araujo R. S. Hungria M. (2016). Accessing inoculation methods of maize and wheat with Azospirillum brasilense. *AMB Expr.* 6:3. 10.1186/s13568-015-0171-y 26759120 PMC4710622

[B26] Gopal M. Gupta A. (2016). Microbiome selection could spur next-generation plant breeding strategies. *Front. Microbiol.* 7:1971. 10.3389/fmicb.2016.01971 28003808 PMC5141590

[B27] Hartman K. van der Heijden M. G. A. Wittwer R. A. Banerjee S. Walser J.-C. Schlaeppi K. (2018). Cropping practices manipulate abundance patterns of root and soil microbiome members paving the way to smart farming. *Microbiome* 6:14. 10.1186/s40168-017-0389-9 PMC5771023 29338764

[B28] Horton M. W. Bodenhausen N. Beilsmith K. Meng D. Muegge B. D. Subramanian S. (2014). Genome-wide association study of Arabidopsis thaliana leaf microbial community. *Nat. Commun.* 5:5320. 10.1038/ncomms6320 PMC4232226 25382143

[B29] Hu L. Robert C. A. M. Cadot S. Zhang X. Ye M. Li B. (2018). Root exudate metabolites drive plant-soil feedbacks on growth and defense by shaping the rhizosphere microbiota. *Nat. Commun.* 9:2738. 10.1038/s41467-018-05122-7 PMC6048113 30013066

[B30] Jarquín D. Crossa J. Lacaze X. Du Cheyron P. Daucourt J. Lorgeou J. (2014). A reaction norm model for genomic selection using high-dimensional genomic and environmental data. *Theor. Appl. Genet.* 127 595–607. 10.1007/s00122-013-2243-1 24337101 PMC3931944

[B31] Johnson N. C. Graham J.-H. Smith F. A. (1997). Functioning of mycorrhizal associations along the mutualism–parasitism continuum. *New Phytologist* 135 575–585. 10.1046/j.1469-8137.1997.00729.x

[B32] Kaminsky L. M. Trexler R. V. Malik R. J. Hockett K. L. Bell T. H. (2019). The inherent conflicts in developing soil microbial inoculants. *Trends Biotechnol.* 37 140–151. 10.1016/j.tibtech.2018.11.011 30587413

[B33] Kecskés M. L. Choudhury A. T. M. A. Casteriano A. V. Deaker R. Roughley R. J. Lewin L. (2016). Effects of bacterial inoculant biofertilizers on growth, yield and nutrition of rice in Australia. *J. Plant Nutr.* 39 377–388. 10.1080/01904167.2015.1016172

[B34] Kiers E. T. Hutton M. G. Denison R. F. (2007). Human selection and the relaxation of legume defences against ineffective rhizobia. *Proc. R. Soc. B: Biol. Sci.* 274 3119–3126. 10.1098/rspb.2007.1187 17939985 PMC2293947

[B35] Lareen A. Burton F. Schäfer P. (2016). Plant root-microbe communication in shaping root microbiomes. *Plant Mol. Biol.* 90 575–587. 10.1007/s11103-015-0417-8 26729479 PMC4819777

[B36] Lebeis S. L. (2014). The potential for give and take in plant-microbiome relationships. *Front. Plant Sci.* 5:287. 10.3389/fpls.2014.00287 24999348 PMC4064451

[B37] Lebeis S. L. Paredes S. H. Lundberg D. S. Breakfield N. Gehring J. McDonald M. (2015). Salicylic acid modulates colonization of the root microbiome by specific bacterial taxa. *Science* 349 860–864. 10.1126/science.aaa8764 26184915

[B38] Liu F. Hewezi T. Lebeis S. L. Pantalone V. Grewal P. S. Staton M. E. (2019). Soil indigenous microbiome and plant genotypes cooperatively modify soybean rhizosphere microbiome assembly. *BMC Microbiol.* 19:201. 10.1186/s12866-019-1572-x 31477026 PMC6720100

[B39] Louca S. Polz M. F. Mazel F. Albright M. B. N. Huber J. A. O’Connor M. I. (2018). Function and functional redundancy in microbial systems. *Nat. Ecol. Evol.* 2 936–943. 29662222 10.1038/s41559-018-0519-1

[B40] Lu T. Ke M. Lavoie M. Jin Y. Fan X. Zhang Z. (2018). Rhizosphere microorganisms can influence the timing of plant flowering. *Microbiome* 6:231. 10.1186/s40168-018-0615-0 30587246 PMC6307273

[B41] Lundberg D. S. Lebeis S. L. Paredes S. H. Yourstone S. Gehring J. Malfatti S. (2012). Defining the core Arabidopsis thaliana root microbiome. *Nature* 488 86–90. 10.1038/nature11237 22859206 PMC4074413

[B42] Magnúsdóttir S. Thiele I. (2018). Modeling metabolism of the human gut microbiome. *Curr. Opin. Biotechnol.* 51 90–96. 10.1016/j.copbio.2017.12.005 29258014

[B43] Malik A. A. Swenson T. Weihe C. Morrison E. W. Martiny J. B. H. Brodie E. L. (2020). Drought and plant litter chemistry alter microbial gene expression and metabolite production. *ISME J.* 14 2236–2247. 10.1038/s41396-020-0683-6 32444813 PMC7608424

[B44] McDonald B. A. Stukenbrock E. H. (2016). Rapid emergence of pathogens in agro-ecosystems: global threats to agricultural sustainability and food security. *Phil. Trans. R. Soc. B* 371:20160026. 10.1098/rstb.2016.0026 28080995 PMC5095548

[B45] Meena K. K. Sorty A. M. Bitla U. M. Choudhary K. Gupta P. Pareek A. (2017). Abiotic stress responses and microbe-mediated mitigation in plants: the omics strategies. *Front. Plant Sci.* 8:172. 10.3389/fpls.2017.00172 28232845 PMC5299014

[B46] Metlen K. L. Aschehoug E. T. Callaway R. M. (2009). Plant behavioural ecology: dynamic plasticity in secondary metabolites. *Plant Cell. Environ.* 32 641–653. 10.1111/j.1365-3040.2008.01910.x 19021888

[B47] Mitter B. Pfaffenbichler N. Flavell R. Compant S. Antonielli L. Petric A. (2017). A new approach to modify plant microbiomes and traits by introducing beneficial bacteria at flowering into progeny seeds. *Front. Microbiol.* 8:11. 10.3389/fmicb.2017.00011 28167932 PMC5253360

[B48] Moose S. P. Mumm R. H. (2008). Molecular plant breeding as the foundation for 21st century crop improvement. *Plant Physiol.* 147 969–977. 10.1104/pp.108.118232 18612074 PMC2442525

[B49] Mueller U. G. Juenger T. E. Kardish M. R. Carlson A. L. Burns K. Edwards J. A. (2019). Artificial microbiome-selection to engineer microbiomes that confer salt-tolerance to plants. *bioRxiv [preprint]* 081521. 10.1101/081521 PMC8631316 34846165

[B50] Mueller U. G. Sachs J. L. (2015). Engineering microbiomes to improve plant and animal health. *Trends Microbiol.* 23 606–617. 26422463 10.1016/j.tim.2015.07.009

[B51] Mutch L. A. Young P. W. (2004). Diversity and specificity of Rhizobium leguminosarum biovar viciae on wild and cultivated legumes. *Mol. Ecol.* 13 2435–2444. 10.1111/j.1365-294X.2004.02259.x 15245415

[B52] Nelkner J. Henke C. Lin T. W. Pätzold W. Hassa J. Jaenicke S. (2019). Effect of Long-Term Farming Practices on Agricultural Soil Microbiome Members Represented by Metagenomically Assembled Genomes (MAGs) and Their Predicted Plant-Beneficial Genes. *Genes (Basel)* 10:424. 10.3390/genes10060424 31163637 PMC6627896

[B53] Nelson M. S. Chun C. L. Sadowsky M. J. (2017). Type IV effector proteins involved in the medicago-sinorhizobium symbiosis. *Mol. Plant. Microbe. Interact.* 30 28–34. 27918247 10.1094/MPMI-10-16-0211-R

[B54] Niu B. Paulson J. N. Zheng X. Kolter R. (2017). Simplified and representative bacterial community of maize roots. *Proc. Natl. Acad. Sci. U.S.A.* 114 E2450–E2459. 28275097 10.1073/pnas.1616148114 PMC5373366

[B55] O’Brien A. M. Jack C. N. Friesen M. L. Frederickson M. E. (2021). Whose trait is it anyways? Coevolution of joint phenotypes and genetic architecture in mutualisms. *Proc. R. Soc. B* 288:20202483. 10.1098/rspb.2020.2483 33434463 PMC7892427

[B56] Parnell J. J. Berka R. Young H. A. Sturino J. M. Kang Y. Barnhart D. M. (2016). From the lab to the farm: an industrial perspective of plant beneficial microorganisms. *Front. Plant Sci.* 7:1110. 10.3389/fpls.2016.01110 27540383 PMC4973397

[B57] Peiffer J. A. Spor A. Koren O. Jin Z. Tringe S. G. Dangl J. L. (2013). Diversity and heritability of the maize rhizosphere microbiome under field conditions. *Proc. Natl. Acad. Sci. U.S.A.* 110 6548–6553. 10.1073/pnas.1302837110 23576752 PMC3631645

[B58] Pérez-Jaramillo J. E. Carrión V. J. Bosse M. Ferrão L. F. V. de Hollander M. Garcia A. A. F. (2017). Linking rhizosphere microbiome composition of wild and domesticated Phaseolus vulgaris to genotypic and root phenotypic traits. *ISME J.* 11 2244–2257. 10.1038/ismej.2017.85 28585939 PMC5607367

[B59] Pérez-Jaramillo J. E. Mendes R. Raaijmakers J. M. (2016). Impact of plant domestication on rhizosphere microbiome assembly and functions. *Plant Mol. Biol.* 90 635–644. 10.1007/s11103-015-0337-7 26085172 PMC4819786

[B60] Pineda A. Zheng S.-J. van Loon J. J. A. Pieterse C. M. J. Dicke M. (2010). Helping plants to deal with insects: the role of beneficial soil-borne microbes. *Trends Plant Sci.* 15 507–514. 10.1016/j.tplants.2010.05.007 20542720

[B61] Ramirez A. M. Stoopen G. Menzel T. R. Gols R. Bouwmeester H. J. Dicke M. (2012). Bidirectional secretions from glandular trichomes of pyrethrum enable immunization of seedlings. *Plant Cell* 24 4252–4265. 23104830 10.1105/tpc.112.105031 PMC3517248

[B62] Ramírez-Flores M. R. Perez-Limon S. Li M. Barrales-Gamez B. Albinsky D. Paszkowski U. (2020). The genetic architecture of host response reveals the importance of arbuscular mycorrhizae to maize cultivation. *eLife* 9:e61701. 10.7554/eLife.61701 33211006 PMC7676867

[B63] Reisberg E. E. Hildebrandt U. Riederer M. Hentschel U. (2013). Distinct phyllosphere bacterial communities on arabidopsis wax mutant leaves. *PLoS One* 8:e78613. 10.1371/journal.pone.0078613 24223831 PMC3818481

[B64] Rey T. Jacquet C. (2018). Symbiosis genes for immunity and vice versa. *Curr. Opin. Plant Biol.* 44 64–71. 29550547 10.1016/j.pbi.2018.02.010

[B65] Ritpitakphong U. Falquet L. Vimoltust A. Berger A. Métraux J.-P. L’Haridon F. (2016). The microbiome of the leaf surface of Arabidopsis protects against a fungal pathogen. *New Phytol.* 210 1033–1043. 10.1111/nph.13808 26725246

[B66] Robertson-Albertyn S. Alegria Terrazas R. Balbirnie K. Blank M. Janiak A. Szarejko I. (2017). Root hair mutations displace the barley rhizosphere microbiota. *Front. Plant Sci.* 8:1094. 10.3389/fpls.2017.01094 28694814 PMC5483447

[B67] Rolli E. Marasco R. Vigani G. Ettoumi B. Mapelli F. Deangelis M. L. (2015). Improved plant resistance to drought is promoted by the root-associated microbiome as a water stress-dependent trait. *Environ. Microbiol.* 17 316–331. 24571749 10.1111/1462-2920.12439

[B68] Roorkiwal M. Jarquin D. Singh M. K. Gaur P. M. Bharadwaj C. Rathore A. (2018). Genomic-enabled prediction models using multi-environment trials to estimate the effect of genotype × environment interaction on prediction accuracy in chickpea. *Sci. Rep.* 8:11701. 10.1038/s41598-018-30027-2 30076340 PMC6076323

[B69] Rovenich H. Boshoven J. C. Thomma B. P. H. J. (2014). Filamentous pathogen effector functions: of pathogens, hosts and microbiomes. *Curr. Opin. Plant Biol.* 20 96–103. 24879450 10.1016/j.pbi.2014.05.001

[B70] Sankaran K. Holmes S. P. (2019). Latent variable modeling for the microbiome. *Biostatistics* 20 599–614. 10.1093/biostatistics/kxy018 29868846 PMC6797058

[B71] Sawers R. J. H. Ramírez-Flores M. R. Olalde-Portugal V. Paszkowski U. (2018). The impact of domestication and crop improvement on arbuscular mycorrhizal symbiosis in cereals: insights from genetics and genomics. *New Phytol.* 220 1135–1140. 29658105 10.1111/nph.15152

[B72] Stringlis I. A. Yu K. Feussner K. de Jonge R. Van Bentum S. Van Verk M. C. (2018). MYB72-dependent coumarin exudation shapes root microbiome assembly to promote plant health. *Proc. Natl. Acad. Sci. U. S. A.* 115 E5213–E5222. 29686086 10.1073/pnas.1722335115 PMC5984513

[B73] Tkacz A. Pini F. Turner T. R. Bestion E. Simmonds J. Howell P. (2020). Agricultural selection of wheat has been shaped by plant-microbe interactions. *Front. Microbiol.* 11:132. 10.3389/fmicb.2020.00132 32117153 PMC7015950

[B74] van der Putten W. H. Bardgett R. D. Bever J. D. Bezemer T. M. Casper B. B. Fukami T. (2013). Plant-soil feedbacks: the past, the present and future challenges. *J. Ecol.* 101 265–276. 10.1111/1365-2745.12054

[B75] Van Deynze A. Zamora P. Delaux P.-M. Heitmann C. Jayaraman D. Rajasekar S. (2018). Nitrogen fixation in a landrace of maize is supported by a mucilage-associated diazotrophic microbiota. *PLoS Biol.* 16:e2006352. 10.1371/journal.pbio.2006352 30086128 PMC6080747

[B76] van Wees S. C. Van der Ent S. Pieterse C. M. (2008). Plant immune responses triggered by beneficial microbes. *Curr. Opin. Plant Biol.* 11 443–448. 10.1016/j.pbi.2008.05.005 18585955

[B77] Varshney G. K. Pei W. LaFave M. C. Idol J. Xu L. Gallardo V. (2015). High-throughput gene targeting and phenotyping in zebrafish using CRISPR/Cas9. *Genome Res.* 25 1030–1042. 10.1101/gr.186379.114 26048245 PMC4484386

[B78] Veach A. M. Morris R. Yip D. Z. Yang Z. K. Engle N. L. Cregger M. A. (2019). Rhizosphere microbiomes diverge among Populus trichocarpa plant-host genotypes and chemotypes, but it depends on soil origin. *Microbiome* 7:76. 10.1186/s40168-019-0668-8 31103040 PMC6525979

[B79] Vorholt J. A. Vogel C. Carlström C. I. Müller D. B. (2017). Establishing causality: opportunities of synthetic communities for plant microbiome research. *Cell Host Microbe* 22 142–155. 10.1016/j.chom.2017.07.004 28799900

[B80] Wagner M. R. Busby P. E. Balint-Kurti P. (2020a). Analysis of leaf microbiome composition of near-isogenic maize lines differing in broad-spectrum disease resistance. *New Phytol.* 225 2152–2165. 31657460 10.1111/nph.16284

[B81] Wagner M. R. Roberts J. H. Balint-Kurti P. Holland J. B. (2020b). Heterosis of leaf and rhizosphere microbiomes in field-grown maize. *New Phytol.* 228 1055–1069. 10.1111/nph.16730 32521050

[B82] Wagner M. R. Lundberg D. S. Coleman-Derr D. Tringe S. G. Dangl J. L. Mitchell-Olds T. (2014). Natural soil microbes alter flowering phenology and the intensity of selection on flowering time in a wild Arabidopsis relative. *Ecol. Lett.* 17 717–726. 10.1111/ele.12276 24698177 PMC4048358

[B83] Wagner M. R. Lundberg D. S. Del Rio T. G. Tringe S. G. Dangl J. L. Mitchell-Olds T. (2016). Host genotype and age shape the leaf and root microbiomes of a wild perennial plant. *Nat. Commun.* 7: 12151. 10.1038/ncomms12151 PMC4945892 27402057

[B84] Wallace J. G. Kremling K. A. Kovar L. L. Buckler E. S. (2018). Quantitative genetics of the maize leaf microbiome. *Phytobiomes J.* 2 208–224. 10.1094/PBIOMES-02-18-0008-R

[B85] Walters W. A. Jin Z. Youngblut N. Wallace J. G. Sutter J. Zhang W. (2018). Large-scale replicated field study of maize rhizosphere identifies heritable microbes. *Proc. Natl. Acad. Sci. U.S.A.* 115 7368–7373. 10.1073/pnas.1800918115 29941552 PMC6048482

[B86] Weinhold A. Karimi Dorcheh E. Li R. Rameshkumar N. Baldwin I. T. (2018). Antimicrobial peptide expression in a wild tobacco plant reveals the limits of host-microbe-manipulations in the field. *eLife* 7:e28715. 10.7554/eLife.28715 29661271 PMC5908438

[B87] Yamada K. Saijo Y. Nakagami H. Takano Y. (2016). Regulation of sugar transporter activity for antibacterial defense in Arabidopsis. *Science* 354 1427–1430. 10.1126/science.aah5692 27884939

[B88] Yeoh Y. K. Dennis P. G. Paungfoo-Lonhienne C. Weber L. Brackin R. Ragan M. A. (2017). Evolutionary conservation of a core root microbiome across plant phyla along a tropical soil chronosequence. *Nat. Commun.* 8:215. 10.1038/s41467-017-00262-8 28790312 PMC5548757

[B89] Zhalnina K. Louie K. B. Hao Z. Mansoori N. da Rocha U. N. Shi S. (2018). Dynamic root exudate chemistry and microbial substrate preferences drive patterns in rhizosphere microbial community assembly. *Nat. Microbiol.* 3 470–480. 29556109 10.1038/s41564-018-0129-3

[B90] Zipfel C. (2014). Plant pattern-recognition receptors. *Trends Immunol.* 35 345–351. 10.1016/j.it.2014.05.004 24946686

[B91] Zipfel C. Oldroyd G. E. D. (2017). Plant signalling in symbiosis and immunity. *Nature* 543 328–336. 28300100 10.1038/nature22009

